# Suppressing Non-Stationary Motion Artefacts in Mobile EEG Using Generalized Eigenvalue Decomposition

**DOI:** 10.3390/s26082440

**Published:** 2026-04-16

**Authors:** Mohammad Khazaei, Khadijeh Raeisi, Patrique Fiedler, Pierpaolo Croce, Filippo Zappasodi, Silvia Comani

**Affiliations:** 1 Department of Neuroscience Imaging and Clinical Sciences, University “G. d’Annunzio” of Chieti–Pescara, 66100 Chieti, Italy; khadijeh.raeisi@unich.it (K.R.); pierpaolo.croce@unich.it (P.C.); filippo.zappasodi@unich.it (F.Z.); 2 Institute of Biomedical Engineering and Informatics, Technische Universität Ilmenau, 98693 Ilmenau, Germany; patrique.fiedler@tu-ilmenau.de; 3 Department of Pediatrics, McGovern Medical School, The University of Texas Health Science Center at Houston, Houston, TX 77030, USA; 4 Institute for Advanced Biomedical Technologies, University “G. d’Annunzio” of Chieti–Pescara, 66100 Chieti, Italy; 5 Behavioral Imaging and Neural Dynamics Center, University “G. d’Annunzio” of Chieti–Pescara, 66013 Chieti, Italy

**Keywords:** automatic artefact removal, motion artefacts, mobile EEG, generalized eigenvalue decomposition

## Abstract

**Highlights:**

**What are the main findings?**

- A GED-based method is proposed for removing highly variable, non periodic motion-related artefacts in mobile EEG data.
- The method uses covariance contrast between rest and motion EEG to identify motion-related artefacts and was validated in two real-world EEG datasets including table tennis gameplay.

**What are the implications of the main findings?**

- GED outperforms ASR in preserving brain signal and suppressing high-amplitude artefacts.
- Unlike ASR, GED avoids introducing high-frequency distortions during denoising.

**Abstract:**

Mobile EEG enables investigating brain activity during real-world behaviour, but remains susceptible to motion artefacts, limiting signal interpretability and the use of advanced analytical techniques. Methods developed for removing motion-related artefacts induced by periodic activity like cycling, walking or juggling showed degraded performance with increasing movement variability and speed. To fill this gap, we developed a method based on generalized eigenvalue decomposition (GED) to identify and suppress highly variable, non-periodic—especially transient—artefacts due to very rapid, free full body movements of different types, as they occur during sports practice. By leveraging the contrast between covariance matrices of artefactual and resting-state EEG segments, this approach isolates motion-related components for removal during multichannel EEG signal reconstruction. The method was validated on two ecological datasets featuring stereotyped head and body movements and dynamic table tennis. Comparison with state-of-the-art technique showed superior performance of our method in terms of signal-to-error ratio (SER), artefact-to-residue ratio (ARR), brain spectral power preservation and computation time. Sensitivity analysis was applied to demonstrate the method’s robustness to parameter changes. These findings highlight the potential of the proposed method as a robust, generalizable approach for motion artefact suppression in mobile EEG, particularly when applied in extreme recording conditions like during active sports activity.

## 1. Introduction

Technological advancements in wearable mobile EEG devices have facilitated the recording of brain activity in diverse real-world environments, including daily life activities, sports and educational contexts, strongly supporting the advancement of our knowledge of the neural mechanisms underlying human social behaviour [1,2,3,4,5,6]. In fact, mobile EEG allows exploration of how cognitive functions, such as attention, stress, and emotional regulation, influence human behaviour and performance in various social contexts. The insights gained from EEG can be leveraged to support mental health and optimize performance by investigating and developing strategies to modulate EEG patterns in the desired direction, e.g., using neurofeedback [7].

While mobile EEG provides valuable insights across various real-world contexts, EEG signals recorded within ecological paradigms are generally affected by reduced signal quality because of free body movements. In fact, in addition to the common physiological and environmental artefacts [8], mobile EEG recordings performed during real-world activities can easily be contaminated by high-amplitude, dynamic, and complex motion-related artefacts [9,10,11] whose suppression remains challenging due to their high amplitude and non-periodic, non-stationary nature. The presence of these artefacts in mobile EEG data recorded during real-world activities (such as sports) prevents the application of advanced analytical methods like functional and effective connectivity or classification of brain states, which require high EEG signal quality to provide reliable results. Dedicated hardware concepts can help reduce the level of artefacts [10], but a substantial amount of motion artefacts can remain in the EEG recordings. Therefore, the availability of well-designed signal processing methods to remove motion-related artefacts and enhance the overall quality of mobile EEG signals is essential for a broad and reliable application of mobile EEG devices in real-world experimental settings, especially those involving free full-body movements.

Conventional methods for artefact removal in EEG, such as bandpass filtering, are inadequate, as considerable frequency content of the artefacts overlaps with that of brain activity, leading to the unintended removal of brain-related signal components [11,12]. To overcome this issue, other methods have been developed to address motion artefacts in mobile EEG recordings. These methods mainly rely on adaptive filtering, blind source separation (BSS), or a combination of these methods [13,14]. Adaptive filters can adjust to changes in artefact characteristics by utilizing a reference signal from an additional sensor. Previous studies have applied this method supported by three-axis head acceleration sensors and channel-tissue impedance measurements [15,16]. However, adaptive filters have a number of drawbacks, including the need for extra sensor hardware, heavy dependence on the quality of the reference signal, and challenges in identifying the nonlinear relationship between the EEG data and the reference signal [8].

BSS methods, such as independent component analysis (ICA), separate brain and non-brain signal components solely using recorded EEG, without the need for additional sensor recordings. In ICA, the fundamental assumption is that EEG signals represent a linear mixture of statistically independent sources corresponding to brain and non-brain components. By removing the non-brain components and reconstructing the EEG signals using the retained components, the denoised EEG signals can be obtained. ICA and its variants are widely used BSS methods for removing artefacts from mobile EEG [17,18,19,20,21,22]. However, one key assumption in ICA for reliable signal separation is the stationarity of the underlying source signals; consequently, the non-stationarity and complex dynamics of motion-related artefacts can be a major issue for the effectiveness of ICA algorithms. ICA-based methods have been proposed to remove motion-related artefacts due to periodic movements like cycling or walking [23,24]. However, these methods were negatively affected by faster walking speeds, especially when walking overground in comparison to treadmill walking, because of higher ground reaction forces and inconsistency of stepping frequency [23]. AMICA is an advanced version of ICA that can automatically learn and apply multiple ICA models to different data segments and has an in-built function to reject bad samples. This makes AMICA particularly effective for data changing over time or containing different types of activity, although it requires more computational power and time because it learns a more complex model than other ICA algorithms [23]. AMICA was tested on EEG data recorded during different movement types (e.g., isometric contractions, treadmill running and ergometer cycling), showing that it performed better or equally well as the Infomax algorithm [25]. More recently, Klug and colleagues [24] tested the effectiveness of AMICA decompositions on eight open-access datasets with varying degrees of motion intensity using varying sample rejection criteria and concluded that movement has a significant negative effect on the decomposition quality. Notably, these conclusions were drawn for datasets affected by—at maximum—periodic movements. Therefore, to date, no ICA algorithm has been proven to perform well in separating motion-related artefacts in EEG data recorded during highly variable, large, fast and free full-body movements, as they can occur during sports activity.

Another crucial challenge in removing motion artefacts by means of ICA is the differentiation between artefactual and brain-related components. In fact, artefactual components are usually identified manually by experts, which restricts the scalability and consistency of this method across studies. Automated ICA-based methods have been developed to detect non-brain signal components [26,27], but they require training on sufficiently similar data to accurately identify and remove artefactual components. Canonical correlation analysis (CCA) is another BSS-based method that can be used to identify artefactual components [28,29]. Like adaptive filters, CCA needs an artefactual reference signal recorded together with the EEG recordings, and both signal types are used as input to CCA to separate artefactual and brain components. In 2023, Gonsisko and colleagues proposed a method that employs CCA in combination with a dual-layer EEG cap, simultaneously recording EEG signals (first layer of sensors) and motion artefacts (second layer of sensors), with the EEG channels mechanically decoupled from the artefact channels, which are used to construct the artefact subspace [29]. While this method can reduce the level of motion artefacts, it relies on the use of specific hardware (the dual-layer cap), which greatly limits its broader applicability and scalability.

Artefact subspace reconstruction (ASR) is a more recent method applied in mobile EEG studies to reduce large amplitude fluctuations generally induced in EEG recordings by body movements [30]. ASR utilizes principal component analysis (PCA) to analyze reference data (clean EEG data) and identify the characteristics of the clean signal components. Based on these characteristics, a threshold for the signal amplitude is set to detect artefactual components separated from EEG recordings. ASR then disregards these artefactual components and reconstructs the EEG signal by keeping only the components within the defined threshold, yielding denoised multichannel EEG signals. Compared to ICA-based methods, ASR has demonstrated its applicability in real-time applications and its effectiveness in removing high amplitude and transient motion artefacts, outperforming ICA- or PCA-based methods in mitigating motion artefacts in EEG recordings [8]. However, ASR contains a free parameter that is used to set the threshold for identifying artefactual components, which must be tuned specifically for each dataset and task [31]. Moreover, ASR involves approximately thirty additional adjustable parameters to consider, although no studies have provided comprehensive guidelines for their optimized tuning yet. Another limitation of ASR is its potential to increase signal power in the high-frequency bands, which may not be desirable in most applications and analyses [32]. More recently, a revised version of ASR with generalized extreme value calibration was successfully employed to remove motion-related artefacts from EEG data recorded during a juggling paradigm [33,34]. However, juggling does not involve abrupt, large, free and full-body movements. Therefore, the generalizability of the advanced version of ASR and its applicability in more demanding contexts, such as during active sports practice, is not demonstrated.

Other methods based on deep learning were introduced for motion-related artefact removal from EEG recordings [26,35,36,37,38]. These approaches have shown promising performance compared to traditional filtering or blind source separation techniques, largely due to their ability to learn complex, non-linear mappings between noisy and clean EEG signals. However, their effectiveness relies heavily on access to large amounts of labelled data spanning a wide range of tasks and artefact types. The datasets most frequently used for training and evaluation of denoising methods are well-suited for physiological artefacts such as ocular (EOG), muscular (EMG), or cardiac (ECG) contamination, and for semi-synthetic benchmarking, but they do not capture the full, coupled biomechanics of artefacts due to naturalistic motion. Factors such as electrode shifts, cap displacement, skin–electrode impedance fluctuations, head acceleration, sweating, and multi-axis body movements are not represented. As a result, while deep learning models can perform well for removing artefacts from EEG signals recorded in constrained conditions, their generalizability remains limited in ecologically valid contexts involving dynamic, free full-body movements.

Our purpose was to fill this gap and develop a method that goes beyond recent advances in motion-related artefact removal and successfully suppresses motion-related artefact also from EEG data recorded during the performance of highly variable, non-periodic free full-body movements characterized by high execution speed and lack of repetitive patterns. The proposed method is based on generalized eigenvalue decomposition (GED), which is a powerful signal decomposition technique that identifies spatial filters maximizing the difference between two covariance matrices—typically representing signal and noise [39]. Leveraging this property, our method decomposes EEG signals into components, discriminates between motion-related (artefactual) and neural (non-artefactual) components, and reconstructs artefact-suppressed EEG by retaining only the non-artefactual components.

To assess the generalizability of our proposed method, hence its capabilities in reducing complex and dynamic motion-related artefacts deriving from EEG recorded in extreme real world conditions, we tested its performance on two distinct datasets of EEG recordings that are affected by very rapid and variable artefacts due to free full-body movements that feature large amplitude variations but do not show any recognizable and repetitive patterns.

Based on the structure of GED, which contrasts the covariance matrix of EEG recorded at rest with the covariance matrix of EEG recorded during motion task performance, and on the nature of the EEG recordings used to validate it, we expect that—when applied to EEG recorded during extreme movement conditions—our proposed method will show superior performance than the most recent version of the actual reference method for motion-related artefact suppression.

## 2. Materials and Methods

**Figure 1:**
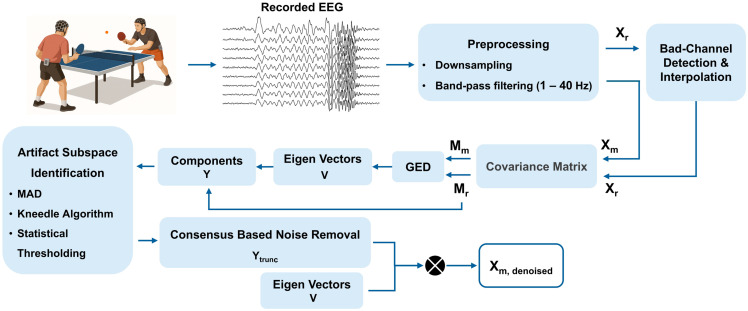
Overview of the proposed EEG denoising method. (X_r_: reference EEG, X_m_: artefactual EEG, GED: generalized eigenvalue decomposition, MAD: median absolute deviation).

The proposed method to enhance EEG signal quality by reducing motion-related artefacts is structured in multiple analytical steps (Figure 1). The first step is the preprocessing of the raw EEG data by means of band-pass filtering, bad-channel detection and interpolation. In the second step, the covariance matrices for the reference EEG (EEG recorded during resting state, hence clean) and artefactual EEG (EEG recorded during the performance of movements) are computed. The third step includes the application of GED to these covariance matrices to derive eigenvectors and components. During the fourth step, the artefactual components are identified using a consensus-based approach and the artefact subspaces are built. During the last step, the denoised EEG is reconstructed by excluding the artefactual components and retaining only the components labelled as non-artefactual. In the following, the datasets used to validate the method and the steps that it comprises are described in more detail.

### 2.1. Datasets

Two different multichannel EEG datasets were used to evaluate the performance of the proposed method: (1) a dataset recorded during the performance of stereotyped head and body movements (stereotyped motion dataset, recorded at the Ilmenau University of Technology, Germany) and (2) a dataset recorded during the performance of cooperative and competitive table tennis games (non-stereotyped motion dataset, recorded at the University of Chieti-Pescara, Italy). Details of each dataset are provided below. All studies complied with the ethical standards outlined in the Declaration of Helsinki and were approved by the responsible local ethics committees. All volunteers signed a written informed consent prior to their participation in the acquisitions. No volunteer had a record of chronic or current neurological, psychological or dermatological pathologies or reported to be under pharmacological treatment.

All EEG recordings were performed using medically certified mobile EEG amplifiers (EE-225 eego, ANT Neuro b.v., Hengelo, The Netherlands). The anti-aliasing filter of the amplifiers had a cut-off at 1/3 of the sampling frequency. The devices integrate active shielding on all EEG channels to reduce susceptibility to cable movement.

#### 2.1.1. Stereotyped Motion Dataset

**Figure 2:**
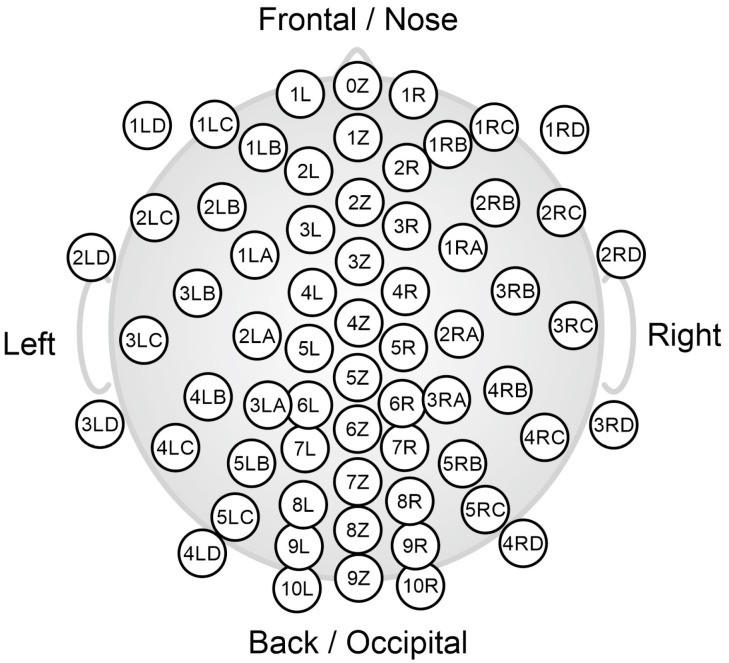
Equidistant channel layout of the 64-channel EEG caps used to acquire EEG data for the Stereotyped and Non-stereotyped motion datasets.

Twenty-eight volunteers (all male, age range 18–65 years, head circumference 54–58 cm) were recruited for this study, which was approved by the Ethics Committee of Technische Universität Ilmenau (Germany, 2023/11/21-4Fied./193.FoA). The study comprised three separate acquisition sessions performed using 64-channel EEG caps with an equidistant channel layout that mounted a different type of electrodes during each acquisition session: commercial gel-based electrodes (waveguard original, ANT Neuro b.v., Hengelo, The Netherlands), commercial dry multipin electrodes [40] (waveguard touch, ANT Neuro b.v.), and novel dry flower electrodes [41]. Figure 2 shows the equidistant channel layout of the 64-channel EEG caps. This study protocol resulted in three EEG recordings per volunteer, each acquired with a different electrode type. Reference and ground channels were always placed at the right and left mastoid, respectively, using self-adhesive hydrogel electrodes. Data were acquired at a sampling rate of 2048 Hz.

During each acquisition session, each volunteer performed blocks of 11 different body movements and 6 different head movements, as shown in the Supplementary Materials. Each type of movement was repeated 20 times in a pseudo-randomized sequence, for a total of 220 body movements and 120 head movements. Movement instructions were presented to the volunteers by displaying an image showing the intended movement on a screen for 3 s, followed by an acoustic and visual trigger to perform the movement. The volunteers had 3 s to perform the indicated movement and return to their initial position prior to proceeding with the next instruction. Three reference eyes-open resting state EEG acquisitions in standing position were also included at: (1) the beginning of the session, (2) between the blocks of body and head movements, and (3) at the end of the session.

#### 2.1.2. Non-Stereotyped Motion Dataset

Thirty-two non-professional right-handed table tennis players who regularly practiced physical activities (all male, right-handed, age range 18–22 years, head circumference 54–59 cm) were recruited for this study. The study was approved by the Ethics Committee of Chieti and Pescara (Italy) (meeting minutes N.06 of 11/03/2021) [42]. EEG hyperscanning acquisitions were performed according to our recently published table tennis protocol, which permits implementing both cooperation and competition within the same experimental framework [42]. During table tennis rallies, EEG signals were recorded simultaneously from each member of the dyad involved by using EEG caps mounting 64 dry flower electrodes [41], arranged in an equidistant layout (same as the one shown in Figure 2), specifically developed to record brain activity during the performance of free body movements. EEG data were recorded at a sampling rate of 1024 Hz.

Table tennis sessions took place in a laboratory room (9 × 6 m) with a professional table tennis table (Cornilleau Table Tennis 500 indoor/outdoor, 274 × 152.5 × 76 cm; 69 kg, Cornilleau SAS, Bonneuil-les-Eaux, France) positioned at the centre of a rectangular area of about 54 m^2^. Players were engaged in a total of 24 table tennis trials of 2.5 min each. Of these trials, 8 trials were played as cooperative table tennis and 16 trials were played as competitive table tennis. During cooperative trials, participants were instructed to keep the ball within the playing area for as long as possible, whereas during competitive trials, they were asked to play as in real table tennis matches, i.e., attempting to outperform their counterpart by scoring more points [42]. The 24 table tennis trials were performed in randomized order. Eyes-open resting state EEG recordings of 6 min duration were included at the beginning and at the end of each EEG acquisition.

### 2.2. Preprocessing

The EEG recordings included in both datasets were downsampled to 256 Hz to reduce computational load during subsequent analysis. Downsampling was done by means of the resample function in MATLAB (Release 2025b), which has a finite impulse response (FIR) anti-aliasing low-pass filter and compensates for the delay introduced by the filter. EEG signals were then high-pass filtered using a zero-phase Hamming-windowed sinc FIR filter (pop_eegfilt function, EEGLAB toolbox) with a cutoff frequency at 1 Hz to mitigate any drift-related frequency components [11]. A default filter order of 768 was used (calculated as 3 × fix (f_s/f_cutoff)), resulting in a transition band of approximately 1 Hz. To eliminate high-frequency artefacts, another zero-phase low-pass FIR filter with a cutoff frequency of 40 Hz was applied. Zero-phase distortion was achieved by forward-backward filtering. For the data recorded at rest, which is used as the reference for denoising, bad channels were identified based on the method proposed by Bailey and colleagues in 2023 [43], which includes the following criteria: (1) large transient amplitude changes that are extreme outliers and likely unrepresentative of the physiological signal components; (2) significant drift; (3) high kurtosis; (4) highly improbable voltage distributions; and (5) log-power log-frequency slopes suggesting muscle activity. Bad channels were removed and interpolated using the spherical spline method implemented in EEGLAB (Release 2025.1.0) [44]. In the following, data termed “raw” refer to EEG data after preprocessing.

### 2.3. Problem Formulation

In the context of EEG recordings contaminated by motion artefacts, the observed signal can be considered as the combination of the signal of interest, which originates from brain sources, and the artefactual components arising from motion-related sources. The EEG ($X\in R^{C\times N}$) can be modelled as:

(1) $$X=A_{b}S_{b}+A_{m}S_{m}$$

where C is the number of EEG channels, N is the number of timepoints, $S_{b}\in R^{n_{b}\times N}$ and $S_{m}\;\in \;R^{n_{m}\times N}$ are random processes representing brain and motion components, and $A_{b}\in R^{C\times n_{b}}$ and $A_{m}\in R^{C\times n_{m}}$ are mixing matrices that model the transfer functions from all possible sources of activity to scalp channels. These mixing matrices can be combined in a global mixing matrix $A\in R^{{(n}_{b}+n_{m})\times N}$ leading to:

(2) X=AS

where $S\in R^{C\times {(n}_{b}+n_{m})}$ indicates all components. These components can be assumed to be independent as they represent different physiological or physical phenomena. The aim of the denoising method is to identify the brain and motion components and separate them to reconstruct EEG signals without motion artefacts.

### 2.4. Generalized Eigenvalue Decomposition

Generalized Eigenvalue Decomposition (GED) is an advanced mathematical method that extends the traditional eigenvalue decomposition. It involves analyzing and contrasting the properties of two matrices by solving the generalized eigenvalue problem, as defined by the equation BV=ρCV, where B and C are matrices, V represents the eigenvectors, and ρ signifies the eigenvalues. Matrix B typically represents a specific signal or phenomenon, such as a data covariance matrix, whereas a matrix C often corresponds to a baseline or control condition. Through this decomposition process, the resulting eigenvectors identify the principal directions where the influence of B is maximized in relation to C, thereby highlighting the distinctive or dominant features between the two matrices. The corresponding eigenvalues then provide a quantifiable measure of the significance of these directions, indicating their relative importance. GED is widely utilized across various scientific and engineering disciplines, including signal processing, where it enhances signal integrity by mitigating artefacts, and statistical pattern recognition, where it facilitates the identification of features that optimally distinguish between different classes or conditions [39].

### 2.5. Motion Artefact Removal Based on Ged

As mentioned in Equation (2), the EEG is constructed from a mixture of brain and motion components. Given that GED highlights the distinctive or dominant features between two matrices, by providing two covariance matrices—one representing clean brain data (without motion artefacts) and the other representing observed data (brain signals corrupted by motion artefacts), GED is able to return the difference between these two matrices, which corresponds to the subspace representing motion artefacts [39]. As shown in Figure 1, EEG is recorded in two conditions: at rest ($X_{r}$), when subjects are asked not to move, and during movement ($X_{m}$), which contains a wide range of motion artefacts. The covariance matrices of these two types of EEG data are used as input to GED, which identifies the components associated with motion artefacts. The covariance matrix for resting state EEG is built using all available resting state EEG recordings. The artefactual components are then excluded from the component space, and clean EEG data (free of motion artefacts) can be reconstructed. Mathematically, the objective of GED is to determine spatial filters that multiply channel timeseries in such a way that the weighted sum of channels maximizes the contrast between the two conditions, in our case between the EEG recorded during resting state (reference EEG free of motion artefacts) and the EEG recorded during the performance of free body movements (hence affected by motion-related artefacts). This goal can be expressed as:

(3) $$\lambda =\frac{{\left\|w^{T}X_{m}\right\|}^{2}}{{\left\|w^{T}X_{r}\right\|}^{2}}$$

where ${\left\|.\right\|}^{2}$ is the norm of the vector. λ is the ratio of the magnitude of the artefactual EEG and the magnitude of the reference EEG, both filtered through w. Equation (3) can be expressed by means of covariance matrices as follows:

(4) $$\lambda =\frac{{\left\|w^{T}X_{m}\right\|}^{2}}{{\left\|w^{T}X_{r}\right\|}^{2}}=\frac{\left(w^{T}X_{m}\right){\left(w^{T}X_{m}\right)}^{T}}{\left(w^{T}X_{r}\right){\left(w^{T}X_{r}\right)}^{T}}=\frac{w^{T}\left(X_{m}X_{m}^{T}\right)w}{w^{T}\left(X_{r}X_{r}^{T}\right)w}$$

where $X_{m}X_{m}^{T}$ and $X_{r}X_{r}^{T}$ can be considered as representation of the covariance matrices, up to the scaling factor of 1/(N−1), of the artefactual and reference EEG, respectively. The expansion of one spatial filter to a set of N spatial filters can be expressed as follows:

(5) $$\Lambda ={\left(W^{T}\left(X_{r}X_{r}^{T}\right)W\right)}^{-1}\left(W^{T}\left(X_{m}X_{m}^{T}\right)W\right)$$

where each column of W is a spatial filter, and each diagonal element of Λ is the corresponding eigenvalue. The first P rows of $Y=W^{T}X_{m}$ span the motion artefact subspace. To separate brain activity from motion artefacts, we define a new vector as follows:

(6) $$\hat{Y}={\left[0,\;\cdots ,\;0,\;y_{P+1},\;\cdots y_{N}\right]}^{T}$$

where artefactual components are set to zero. Now, the estimated brain activity can be obtained as follows:

(7) $${\hat{X}}_{m}=W^{-T}\hat{Y}$$

In practice, the covariance matrices for the reference and artefactual EEG are calculated, for each of these EEG signals, by segmenting the signal into windows of 1 s, calculating the covariance matrix for each window, and finally calculating the average covariance matrix by applying the Riemannian geometric averaging [45,46]. The two average covariance matrices (one for the reference EEG and one for the artefactual EEG) are used as input to GED. In the operational mode, the pre-computed, subject-specific $X_{r}X_{r}^{T}$ matrix is applied to the EEG data and remains static throughout the experiment.

### 2.6. Identifying Artefact Subspace

To identify artefactual components, we employed three complementary eigenvalue-based methods. Only the components that are identified as artefactual by all three approaches were included in the artefact subspace. The first method used to detect artefactual components is a robust outlier criterion based on the median absolute deviation (MAD) [47]. The second method, developed by Satopää and colleagues [48], determines the elbow point in the eigenvalue distribution, with components beyond this point classified as artefactual. This method has the advantage of being simple and able to handle diverse curve types without requiring user input or parameter tuning, making it particularly effective for unsupervised tasks. The third method is based on the construction of an empirical null distribution of the largest eigenvalues obtained from resting-state EEG data, and subsequent comparison between this distribution and the eigenvalues of the separated components to categorize them as either artefactual or non-artefactual. The statistical comparison is performed at 95% confidence level [39].

### 2.7. Evaluation Criteria

An ideal denoising method should preserve uncontaminated EEG originating from brain activity while effectively suppressing artefactual components. To evaluate this balance between signal preservation and artefact removal, we employed three metrics: the signal-to-error ratio, the artefact-to-residue ratio, and the spectral power.

*Signal-to-Error Ratio (SER):* The signal-to-error ratio is a metric used to assess potential distortion of the clean EEG by the proposed denoising method [49]. SER is calculated from the EEG marked as artefact-free. In both datasets, artefact-free EEG corresponds to EEG signals that were recorded at rest. To compute SER for each EEG channel, the expected value of the squared signal in the “raw” data across all artefact-free recordings (x*_i_*) is obtained, and this value is divided by the expected value of the squared signal removed by the denoising method across the artefact-free recordings (${\hat{d}}_{i}$). It is important to note that the “raw” EEG used for SER calculation is obtained after preprocessing (cp. Section 2.2). To derive a single SER measure for the entire denoised recording, the SER values from individual EEG channels were combined using a weighted average, with the weighting based on the proportion of artefact contributed by each channel relative to the total artefact across all EEG channels ($p_{i}$). The mathematical expression for SER is as follows:

(8) $${SER}_{i}=10{log}_{10}\frac{E\left\{x_{i}^{2}\right\}}{E\left\{{\hat{d}}_{i}^{2}\right\}},\;\;\;\;\;\;\;\;\;\;\;\;i=1,\;\ldots ,\;N$$

(9) $$SER=\sum_{i=1}^{N}p_{i}.{SER}_{i}$$

where *N* is the number of EEG channels and $p_{i}$ is calculated as follows:

(10) $$p_{i}=\frac{E\left\{{\left(x_{i}\right)}^{2}\right\}\;\left(noisy\;segments\right)-\;E\left\{{\left(x_{i}\right)}^{2}\right\}\;(clean\;segments)}{\sum_{i=1}^{N}\left(E\left\{{\left(x_{i}\right)}^{2}\right\}\;\left(noisy\;segments\right)-\;E\left\{{\left(x_{i}\right)}^{2}\right\}\;(clean\;segments)\right)}$$

High values of SER are anticipated when the denoising method leaves the artefact-free recordings of the EEG data undistorted, thus indicating a good performance.

*Artefact-to-Residue Ratio (ARR):* ARR is a metric that measures how effectively an artefact removal algorithm has suppressed undesirable noise [49]. ARR is calculated from the EEG segments marked as artefactual. In both datasets, artefact-free segments correspond to EEG signals that were recorded at rest. Like for SER, ARR is first calculated on individual EEG channels by obtaining the expected value operator of the square of the removed artefact ($d_{i}$) divided by the expected value of the square of the total signal from the artefactual segments (${\hat{x}}_{i}$) in the “raw” data (x*_i_*). To derive a single value for each recording, the individual EEG channel values were combined via weighting, similarly to SER. ARR is a valid metric when artefacts are significantly larger in amplitude compared to the artefact-free EEG. A high ARR value indicates better artefact reduction, i.e., that more artefacts are removed from the artefactual EEG segments. Again, the “raw” EEG used for ARR calculation is obtained after preprocessing (cp. Section 2.2). ARR is mathematically expressed as follows:

(11) $${ARR}_{i}=10{log}_{10}\frac{E\left\{d_{i}^{2}\right\}}{E\left\{{\left(d_{i}-{\hat{d}}_{i}\right)}^{2}\right\}},\;\;\;\;\;\;\;\;\;\;\;\;i=1,\;\ldots ,\;N$$

where $d_{i}$ is the artefact and ${\hat{d}}_{i}$ is the estimated artefact. As $d_{i}$ is not available for real data, ARR is computed by substituting $d_{i}$ by $x_{i}$ in Equation (11). This approximation is valid for artefacts exhibiting transient high amplitudes.

*Power:* The power of the EEG calculated on the high frequency band (30–128 Hz) is used to measure whether a denoising method introduces high frequency artefacts, as observed, e.g., for the ASR method [30]. Here, the EEG power spectral density (PSD) within the mentioned frequency band was calculated using Welch’s method on EEG windows of 256 samples, with a 50% overlap between consecutive windows and a resolution of 0.1 Hz. The reduction or increase in high-frequency noise due to the denoising method is assessed by calculating the difference between the power in the EEG signals before and after denoising (with our proposed GED-based method and the reference method, i.e., ASR at various cutoff values).

*Sensitivity analysis:* To assess the robustness of our GED-based method with respect to parameter changes, we implemented a sensitivity analysis using the EEG recordings of the non-stereotyped motion dataset. We performed a comparative analysis of the SER and ARR values obtained for GED and the three variants of ASR (ASR10, ASR20 and ASR30) using windows of different durations (0.5 s, 1 s, 1.5 s, 2 s).

*Computation time:* The suitability of our GED-based method for online applications was validated by calculating the computation times of calibration and processing, as well as the per-window latencies. We used the EEG data included in the non-stereotyped motion dataset because they were recorded during real table tennis trials, hence present very large and irregular motion-related artefacts. Computation times were calculated as averages on the 64 channels of the EEG cap, with EEG signals downsampled to 256 Hz, using 20 repetitions on non-overlapping windows of 1 s length. The same computation times were calculated for ASR10 on the same EEG data for comparison. In both cases, we used a computer with an Intel Core(TM) i7-14700 K (3.40 GHz) processor, 64 GB RAM, and a 64-bit operating system.

### 2.8. Statistical Analysis

Statistical analyses were performed to compare the denoising performance of the proposed GED-based method against three ASR variants (ASR10, ASR20, ASR30). We used the clean_rawdata plugin for ASR (version 2.2) with its default parameters for all internal settings, except for the cutoff threshold. Following recent empirical evidence [50] and the recommendations in the EEGLAB documentation (Makoto’s Preprocessing Pipeline), we chose three values for the cutoff threshold: 10, 20 and 30:

- ASR 10 was chosen to represent a more aggressive cleaning strategy, which is often hypothesized to be necessary for high levels of motion contamination, as found in mobile settings like sports.
- ASR 20 was selected as the recommended “gold standard” for adult EEG data to avoid inadvertent removal of genuine brain signals.
- ASR 30 served as a conservative baseline to ensure that our comparisons accounted for the highest level of physiological signal preservation.

Given that all denoising methods were applied to the same sets of EEG recordings, paired statistical testing was performed using the Wilcoxon signed-rank test. For the stereotyped motion dataset, the analysis included 84 observations (28 volunteers × 3 acquisition sessions each). For the non-stereotyped motion dataset, the analysis included 32 observations (32 participants). Significance level was set at *p* < 0.05. All statistical procedures were implemented in MATLAB (Release 2025b).

## 3. Results

**Figure 3:**
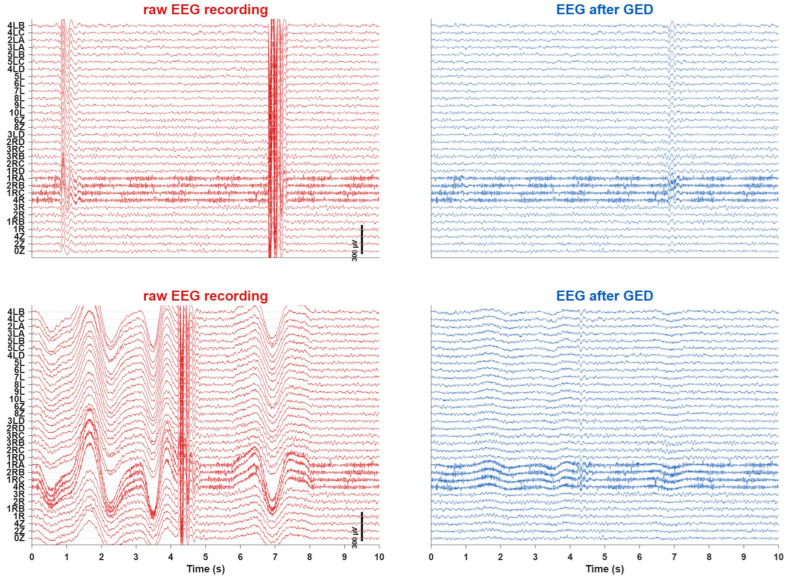
Examples of EEG recordings containing motion artefacts and their corresponding denoised versions in the non-stereotyped motion dataset. The (**left** panels) display EEGs contaminated with motion artefacts, whereas the (**right** panels) show the denoised EEGs obtained after applying our GED-based method. The (**upper** panels) refer to EEG recorded during cooperative table tennis, whereas the (**lower** panels) refer to EEG recorded during competitive table tennis. Channels 1RA, 2RB,1RC and 4R show bad sensor-scalp contact that are subsequently removed and interpolated using the spherical spline method.

Figure 3 shows two example epochs of EEG before and after the application of the proposed GED-based method (subject MRGDVD1, dyad 10, table tennis rally, recording date 31 March 2025). The raw EEG (in red) exhibits motion artefacts that obscure the underlying brain activity. After applying GED (in blue), the artefacts due to movement are significantly reduced. Please note that the lower panels refer to EEG recorded during competitive table tennis, which is much more dynamic than cooperative table tennis (upper panels). Channels 1RA, 2RB,1RC and 4R show bad sensor-scalp contact that is not removed by our proposed method, but—as described in Section 2.2 Preprocessing—need to be removed and interpolated using the spherical spline method.

**Figure 4:**
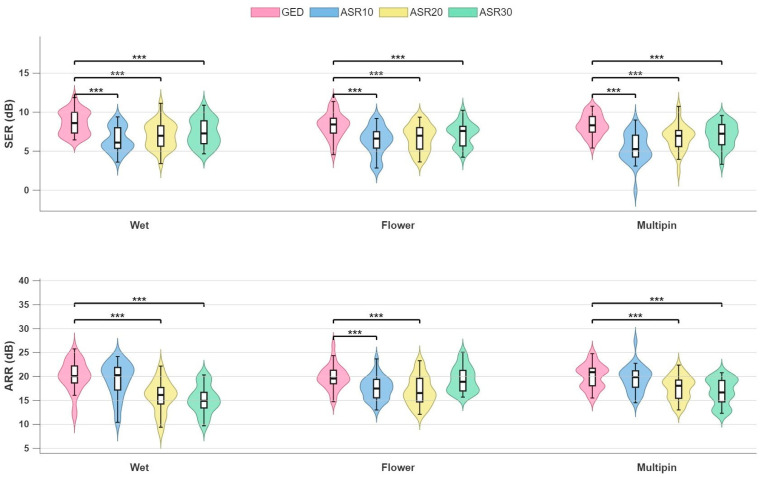
Comparative analysis of EEG denoising methods on the stereotyped motion dataset across three EEG cap types using SER (**top** panel) and ARR (**bottom** panel). The white box refers to the interquartile range (IQR) with the median value indicated by the black dash. Statistically significant differences are indicated by asterisks (Wilcoxon signed-rank test, *p* < 0.001) (SER: signal-to-error ratio, ARR: amplitude-to-residue ratio, GED: generalized eigenvalue decomposition, ASRx: artefact subspace reconstruction with a cut-off of x).

**Figure 5:**
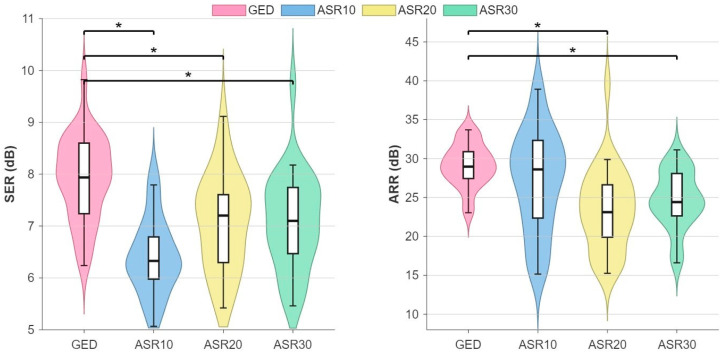
Comparative analysis of EEG denoising methods on the non-stereotyped motion dataset using SER (**left** panel) and ARR (**right** panel). The white box refers to the interquartile range (IQR) with the median value indicated by the black dash. Statistically significant differences are indicated by asterisks (Wilcoxon signed-rank test, *p* < 0.05) (SER: signal-to-error ratio, ARR: amplitude-to-residue ratio, GED: generalized eigenvalue decomposition, ASRx: artefact subspace reconstruction with a cut-off of x).

The values of SER and ARR obtained on both datasets demonstrated the overall superior performance of our proposed GED-based method vs. ASR in mitigating motion-related artefacts while preserving the integrity of brain activity content in the EEG signals. When assessing the potential distortion of the EEG signals obtained after removal of motion-related artefacts, we observed that the SER values for GED were always significantly higher than those obtained for ASR across all EEG cap types and for both datasets (Figure 4 and Figure 5).

For the Stereotyped motion dataset, we obtained the following SER values for the different cap types (Figure 4, upper panels). For the Wet cap, GED achieved a median SER of 8.8 [IQR 2.1] dB, significantly exceeding ASR10 (6.1 [1.8] dB; *p* = 0.001), ASR20 (7.5 [2.6] dB; *p* = 0.001), and ASR30 (7.7 [2.1] dB; *p* = 0.001). For the Flower cap, SER for GED (8.2 [2.3] dB) was significantly higher than for ASR10 (6.2 [3.0] dB; *p* = 0.001), ASR20 (6.5 [3.8] dB; *p* = 0.001), and ASR30 (7.5 [2.5] dB; *p* = 0.001). A similar pattern was observed for the Multipin cap, where GED significantly outperformed all ASR variants (GED: 8.5 [2.1] dB; ASR10: 5.5 [2.2] dB, *p* < 0.001; ASR20: 6.6 [2.5] dB, *p* = 0.001; ASR30: 6.7 [2.2] dB, *p* = 0.001). On all cap types, ASR10 yielded the lowest SER values, consistently with the well-documented over-cleaning behaviour of aggressive threshold settings that suppress not only artefactual signal components but also genuine neural signal variance, hence distorting true brain signals.

Also, for the Non-stereotyped motion dataset (Figure 5), our GED-based method consistently achieved higher signal preservation than all three ASR variants. Across the full dataset, GED yielded a median SER of 8.1 [IQR: 1.2] dB, significantly exceeding ASR10 (6.5 [1.4] dB; *p* =0.041), ASR20 (7.3 [1.8] dB; *p* = 0.031), and ASR30 (7.1 [1.5] dB; *p* = 0.049). Also, for this dataset, ASR10 yielded the lowest SER values.

The ARR results revealed a more nuanced pattern across cap types (Stereotyped motion dataset, Figure 4, lower panels). For the Wet cap, ARR for our GED-based method (20.5 [2.2] dB) was similar to that for ASR10 (20.3 [3.0] dB, *p* = 0.07), whereas it was significantly superior to ARR for ASR20 (15.3 [3.1] dB, *p* < 0.001) and ASR30 (14.9 [2.8] dB, *p* < 0.001). A similar pattern was observed for the Multipin cap, with ARR for our GED-based method (21.8 [4.5] dB) being similar to that for ASR10 (19.9 [3.6] dB) but significantly superior to that for ASR20 (17.3 [3.3] dB, *p* < 0.001) and ASR30 (16.7 [4.6] dB, *p* < 0.001). For the Flower cap, instead, ARR for our GED-based method (19.2 [3.9] dB) was not significantly different from that obtained for ASR30 (17.6 [3.8] dB, *p* = 0.1), whereas it was significantly higher than ARR for ASR10 (17.4 [2.3] dB, *p* < 0.001) and ASR20 (16.5 [3.3] dB, *p* < 0.001).

The ARR values for the Non-stereotyped motion dataset were overall higher than those obtained from the Flower cap recordings of the Stereotyped motion dataset. In particular, our GED-based method achieved a median ARR of 29.5 [4.1] dB, significantly outperforming ASR20 (23.5 [7.4] dB; *p* = 0.047) and ASR30 (24.0 [9.6] dB; *p* = 0.038). In contrast, the difference between ARR for our GED-based method and for ASR10 (28.8 [12.4] dB) did not reach statistical significance (*p* = 0.14). This null result for ASR10 can be due to its high inter-subject variability, reflected in the substantially larger IQR compared to the IQR of both GED and the other ASR variants.

**Figure 6:**
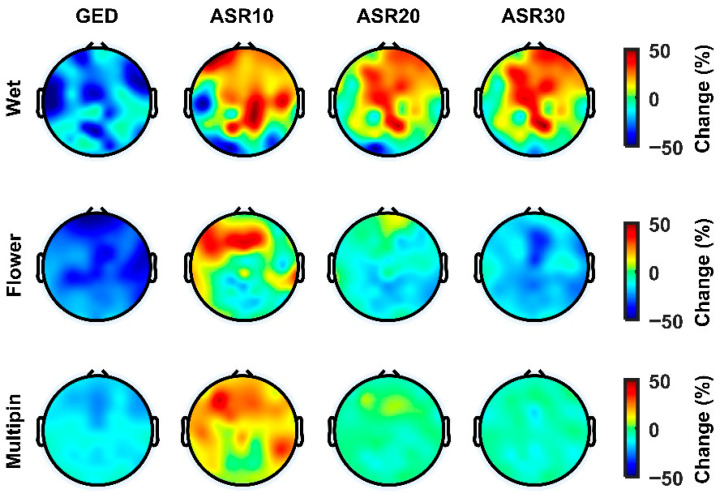
Topographical maps depicting the relative change in high-frequency power (>30 Hz) of EEG signals after application of different denoising methods to the stereotyped motion dataset across three EEG cap types: Wet, Flower, and Multipin. The red colour shows the increase in relation to the raw signal, and the blue colour shows the decrease. (GED: generalized eigenvalue decomposition, ASRx: artefact subspace reconstruction with a cut-off of x).

**Figure 7:**
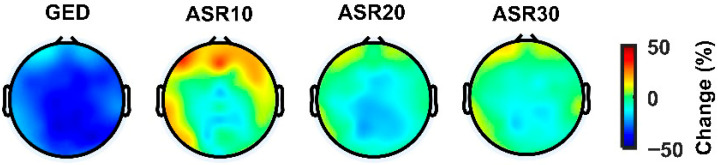
The relative change in high-frequency power (>30 Hz) of EEG signals after application of different denoising methods to the non-stereotyped motion dataset. The red colour shows the increase in relation to the raw signal, and the blue colour shows the decrease. (GED: generalized eigenvalue decomposition, ASRx: artefact subspace reconstruction with a cut-off of x).

The topographical analysis of the PSD indicates that the GED-based method consistently mitigated high-frequency (>30 Hz) power across the entire scalp, as shown by widespread blue regions, suggesting an effective artefact reduction without the introduction of unwanted high-frequency noise (Figure 6 and Figure 7). Conversely, the ASR variants, particularly ASR10, exhibited localized increases in the high-frequency power (red regions), especially in the fronto-central areas. In comparison, ASR20 and ASR30 demonstrated moderate effectiveness in reducing high-frequency power, with only slight reductions, less impactful than the proposed GED-based method and consistent with the more conservative threshold setting.

**Figure 8:**
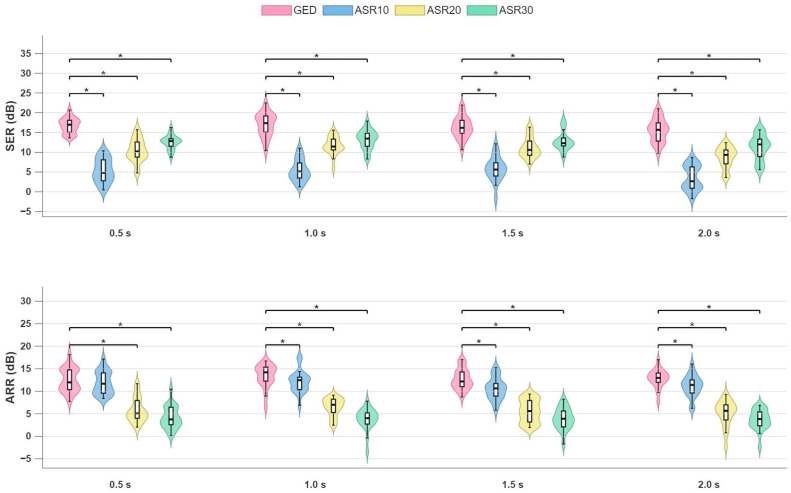
Results of the sensitivity analysis. Four windows of different lengths (0.5 s, 1 s, 1.5 s and 2 s) were used for both the GED-based method and all ASR variants. Asterisks indicate statistically significant differences (Wilcoxon signed-rank test, *p* < 0.05).

Figure 8 shows the results of the sensitivity analysis. Significant differences were observed for SER and ARR between our GED-based method and all ASR variants, similarly to the results obtained for the analysis using the default window lengths for both methods (see Figure 4 and Figure 5). Notably, no significant differences were observed for the SER and ARR values obtained with our GED-based method using different window lengths (GED 0.5 s: SER 16.9 [IQR: 2.2] dB, ARR 11.9 [IQR: 3.0] dB; GED 1 s: SER 16.8 [2.3] dB, ARR 14.9 [2.5] dB; GED 1.5 s: SER 16.1 [2.2] dB, ARR 12.2 [2.5] dB; GED 2 s: SER 15.6 [2.4] dB. ARR 12.9 [1.8] dB; *p* > 0.5), demonstrating the robustness of our method to parameter changes.

The analysis of computation times showed that our GED-based method and ASR10 had similar calibration times (GED: 0.28 ± 0.02 s; ASR10: 0.32 ± 0.03 s), with the advantage of GED over ASR that its calibration phase is one-time per subject. In fact, the resting-state covariance matrix is computed once, stored, and reused for all subsequent trials without re-computation. Conversely, calibration for ASR must be re-run whenever the cutoff parameter is changed. Computation times for the processing phase showed that the GED-based method is much faster than ASR10, with the speed difference between the two methods increasing with the EEG recording length (Table 1). In fact, for EEG recordings of 1 min, the GED-based method is 3.5 times faster than ASR, but for EEG recordings of 10 min, the GED-based method is 4.3 times faster than ASR. Notably, the standard deviation of computation time for the GED-based method is also approximately one-third of the standard deviation of computation time for ASR. This difference arises from the fact that GED applies a single generalized eigenvalue decomposition to each non-overlapping window, whereas ASR applies PCA-based reconstruction to every sliding window. The per-window latencies were far below the window length for both methods (about 0.30 ms).

## 4. Discussion

Recent developments in neuroscience include the study of brain dynamics during the performance of daily life activities, during sports practice and during interpersonal interactions. These new ecological applications of EEG raised the question of relevant EEG contamination with artefacts due to free movements in the environment. Several approaches were proposed to reduce motion-related artefacts in the EEG. Very recently, a method based on AMICA decompositions and a revised version of ASR with generalized extreme value calibration were proposed and tested on EEG datasets recorded during periodic movements like walking or jogging [24] and during cooperative juggling [24,33,34]. Good results were obtained on these activities, which are characterized by periodic patterns, quasi-stationary amplitudes and relatively low movement speed. However, when tested on more rapid movements with larger amplitude variations, their performance substantially decreased [24,33,34,51].

We intended to go beyond the practical limitation of EEG recorded during moderate physical activity and design a method able to effectively reduce motion-related artefacts also from EEG recorded in very demanding conditions, such as during the performance of very rapid, non-stereotyped, non-periodic body movements with large amplitude variations and no steady patterns, as it generally occurs in sports practice. Therefore, we designed a method based on GED because, to identify the subspace representing motion artefacts, GED calculates the difference between two covariance matrices, one calculated for the resting state EEG—which acts as a reference EEG signal—and the other for the EEG recorded during activity. However, in real-world mobile EEG scenarios and especially for long EEG recordings, physiological states may change over time, which could cause a drift problem where the resting-state covariance matrix no longer accurately represents the brain dynamics during the activity. To overcome this problem and provide GED with resting state EEGs that are as representative as possible of brain states over time, we employed EEG datasets including multiple resting state EEGs per recording. One might claim that the use of multiple EEGs at rest is a limitation to the applicability of this method. However, it is common and good practice in neuroscience to register multiple resting state EEGs, and we think that, even in cases when they were not planned, it would be a low cost to pay for reaching an effective reduction in motion contamination in the EEG recorded during activity.

To provide generalizable results, the performance of our proposed method was evaluated on two distinct datasets: one containing EEG data recorded with EEG caps mounting three different electrode types and including triggered stereotyped body and head movements, and another including EEG data acquired during table tennis gameplay, hence involving gross, rapid, non-stereotyped free full-body movements that occur with random sequences. To our knowledge, this is the first time that a method to remove motion-related artefacts has been tested on EEG data recorded in such complex environmental conditions and using different electrode types.

The performance of our GED-based method was compared with that of the advanced version of ASR, which is considered the state-of-the-art method for removing motion artefacts. In fact, ASR was effectively employed in EEG data recorded during juggling, an experimental condition similar to the one that we addressed, although juggling involves smooth and periodic movements of regular amplitude [33,34]. We compared the performance of these two methods in terms of SER and ARR, two metrics that are recognized to provide reliable evaluations of the potential distortion of the clean EEG due to the denoising process (SER) and of a method’s effectiveness in suppressing undesired artefacts (ARR) [49]. Results revealed that the proposed GED-based method had an overall superior performance than ASR in attenuating non-stereotyped large amplitude fluctuations due to body movements while better preserving true brain signals.

The SER values for our proposed method were significantly higher than those obtained for all three variants of ASR on both datasets. No substantial differences in behaviour were observed across the EEG caps, testifying to the equivalence of the three electrode types in terms of EEG signal quality and the independence of our method on the type of EEG system used. It is also worth noting that the median SER obtained for our GED-based method on table tennis EEG data was very close to the SER values obtained for the stereotyped motion dataset, and that in both cases the interquartile ranges were very low. Given the much higher amount and more variable nature of the motion-related artefacts affecting the EEG recorded during table tennis, these results can be considered as indirect proof of the robustness of our method, whose performance is not affected by increased movement speed or amplitude variations.

In general, our GED-based method also showed better suppression of motion-related artefacts than ASR. In fact, ARR values for GED were higher than those for the less aggressive variants of ASR (i.e., ASR20 and ASR30), whereas no significant differences were found with the most aggressive variant of ASR (i.e., ASR10), except for the Flower cap EEG recordings of the Stereotyped motion dataset. However, the ARR results for ASR10 on the most demanding dataset to denoise—i.e., the dataset of table tennis EEG recordings, showed that, although the median ARR of ASR10 and GED were similar, the distribution of ARR values for ASR10 was much more dispersed, indicating that the aggressive ASR10 threshold produced highly inconsistent outcomes across subjects, with some EEG recordings benefiting from near-complete artefact removal while others remained affected by substantial artefact contamination. This observation is consistent with the finding that ASR10 yielded the lowest SER values across all conditions, reflecting the well-documented over-cleaning behaviour of aggressive ASR thresholds, with consequent distortion of the underlying brain signals.

The superior performance of our GED-based method in removing motion-related artefacts from EEG signals can be attributed to the hypothesis-driven, contrast-enhancing nature of GED, which favours a better separation of artefacts from genuine physiological components compared to state-of-the-art methods. GED was designed with the goal of preserving the underlying neural signal while minimizing the impact of artefacts. Unlike unsupervised methods like PCA, which merely capture the largest variance, GED utilizes a clear separation criterion that permits it to more effectively distinguish artefacts from physiological brain signals based on their statistical differences [39]. This targeted contrast allows GED to retain low-variance, clean EEG features that might otherwise be overshadowed or altered by high-amplitude artefacts in methods that rely solely on variance, such as PCA or ASR.

Our study highlights another remarkable distinction in how GED and ASR handle high-frequency EEG signal components (>30 Hz). Specifically, we demonstrated that our GED-based method achieves spatially uniform signal reduction, consistent with effective artefact removal without introducing high-frequency artefacts during large-amplitude artefact rejection. In contrast, all ASR variants introduce topographically specific distortions that vary substantially across cap types. The spatial heterogeneity of ASR-induced changes—particularly the coexistence of strong positive and negative regions—may indicate that ASR selectively modifies the spectral content of the EEG in a spatially non-uniform manner, which may compromise the validity of subsequent topographic analyses. We believe that our GED-based method had a superior performance because the output of a spatial filter like GED is dominated by the frequency band with the most power, and EEG data generally exhibit a 1/f power distribution. As a result, spatial filters work effectively only for low-frequency signals. High-frequency signals are poorly decomposed and remain correlated. Rejecting these correlated components can lead to a counterintuitive increase in signal power due to de-cancellation [31,32]. The contrast-enhancing nature of the source decomposition method implemented in GED may contribute to its ability to better manage high-frequency ranges, potentially enabling GED to more effectively separate brain and artefactual components due to its supervised decomposition. Additionally, the results of our study suggest that the level of high-frequency artefact introduced by ASR in the processed EEG signals may depend on the chosen cut-off parameter. In fact, worse results were obtained for ASR10, which is the ASR variant featuring the best results in terms of artefact removal. A comprehensive simulation study with known ground truth would be required to draw a definitive conclusion on the dependence of ASR on the value of the cut-off parameter, but this assessment is out of scope in the present study.

All together, these findings underscore that our proposed GED-based method is superior to the latest version of ASR in effectively suppressing complex and non-stationary motion artefact contamination while preserving the integrity of EEG signals recorded during dynamic free full-body movements. These results are particularly relevant because the effectiveness of the GED-based method in mitigating motion-related artefacts was assessed on EEG signals recorded in an extremely challenging scenario, i.e., EEG data collected during table tennis gameplay. In fact, table tennis players continuously perform rapid and multidirectional arm swings, head movements, and whole-body shifts that generate a wide range of motion-related artefacts that are rapid, non-stereotyped, of highly variable amplitude, repetitive but not periodic. Such complex artefacts are critical to be identified and removed for any denoising method that also aims at preserving physiological EEG components. The robustness of our GED-based method was also confirmed by the results of the sensitivity analysis, demonstrating how the proposed method is essentially insensitive to parameter changes. The very rapid computation times obtained both for short and long EEG recordings, due to the method’s architecture, highlighted the suitability of our method for online processing applications, a key feature in ecological experimental contexts of social neuroscience and human–machine interactions aiming for direct feedback.

Notwithstanding the good results, we should mention that there is still room for improving the proposed GED-based method. Currently, our procedure for identifying the artefact subspace is based on the consensus among three methods, but it could be enhanced by developing machine learning models that can extract relevant features from the topography of components and component activities, as proposed in previous studies [26,27]. Future research could focus on refining our approach by using adaptive criteria tailored to the specific EEG datasets and artefact profiles under investigation, and on a more general assessment of the effectiveness of our GED-based method on EEG signals recorded in other movement-based settings [52,53,54].

## 5. Conclusions

In this study, we introduced and validated a supervised GED-based method to suppress highly variable, rapid, non-stereotyped and non-periodic motion-related artefacts from mobile EEG recordings. By leveraging the statistical contrast between artefactual and reference conditions, this method proved to be superior to ASR in identifying and reducing motion-related components without compromising brain signal integrity, including high-frequency components that are often distorted by ASR and other denoising methods.

The evaluation across two ecologically valid datasets of EEG recordings, including stereotyped movement artefacts and motion-related artefacts due to naturalistic table tennis gameplay, underscored the robustness and generalizability of our proposed method in dynamic, movement-intensive contexts. These findings, together with the very low computation times, highlight our GED-based method as a promising solution for advancing the applicability of mobile EEG in ecological social and sports neuroscience, as well as in human–machine applications, where artefactual contamination due to free body movements poses a major obstacle to reliable interpretation of the brain signals.

## Figures and Tables

**Table 1:** Mean ± SD processing time for GED and ASR10 as a function of the EEG recording duration during the processing phase (per EEG trial, varying duration).

EEG Recording Duration (min)	GED Processing Time (s)	ASR Processing Time (s)
1	0.020 ± 0.002	0.070 ± 0.007
2	0.040 ± 0.003	0.150 ± 0.009
5	0.090 ± 0.007	0.370 ± 0.026
10	0.180 ± 0.014	0.770 ± 0.021

## Data Availability

The original data presented in the study are openly available (DOI:10.5281/zenodo.17471747). The code of our proposed GED-based method is published in Zenodo: https://zenodo.org/records/17471748 (accessed on 30 October 2025).

## Supplementary Materials

The following supporting information can be downloaded at: https://www.mdpi.com/article/10.3390/s26082440/s1, File S1: Description of the study protocol used for the acquisition of the EEG recordings for the stereotyped motion dataset.
