# Supplementary material for: Suppressing Non-Stationary Motion Artefacts in Mobile EEG Using Generalized Eigenvalue Decomposition
Source: Sensors (Basel). 2026 Apr 16;26(8):2440. doi: 10.3390/s26082440 (PMC13119521; doi:10.3390/s26082440)
Supplement: Supplementary file 1 [file sensors-26-02440-s001.zip › sensors-4215682-supplementary.pdf]

Supplementary Materials S1

Description of the study protocol used for the acquisition of the EEG recordings for the stereotyped motion dataset.

Experimental protocol

During each acquisition session, each volunteer performed blocks of 11 different body movements or 6 head movements, according to the following list. The movements aimed at simulating dominant movements during table tennis. The pictures included in the following are the same used to trigger movement execution during EEG data acquisition.

Body movements (20 repetitions for each of the 11 movement types, in pseudo-randomized sequence):

SQUAT	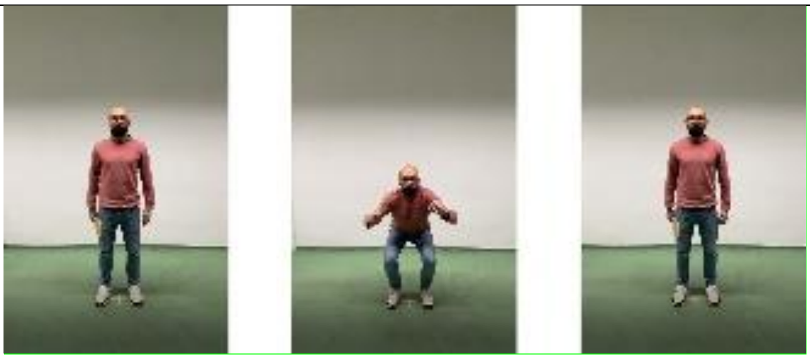
SIDE STEP LEFT	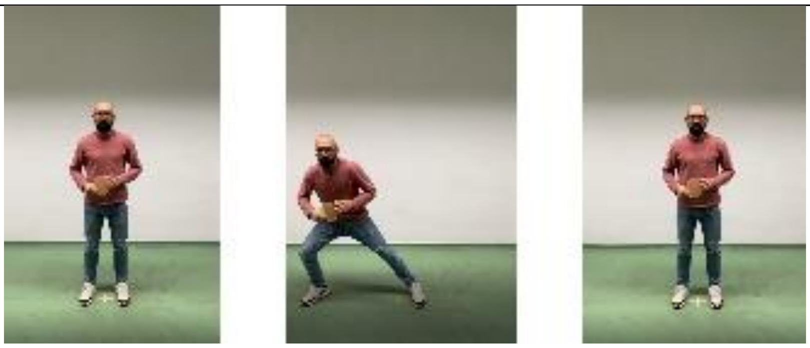
SIDE STEP RIGHT	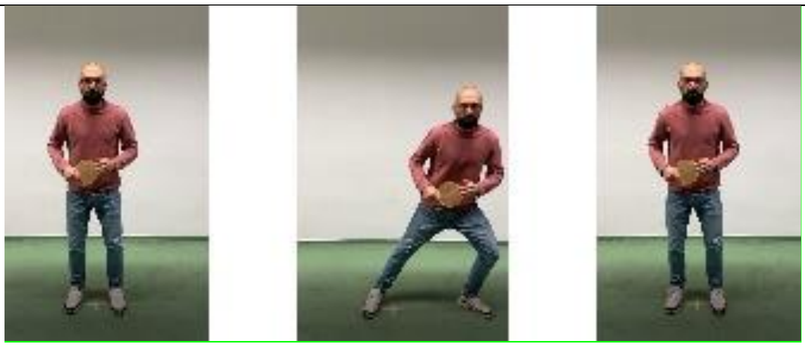

FORWARD STEP LEFT	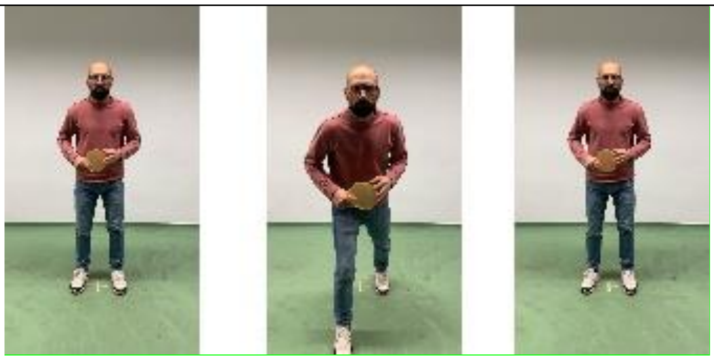
FORWARD STEP RIGHT	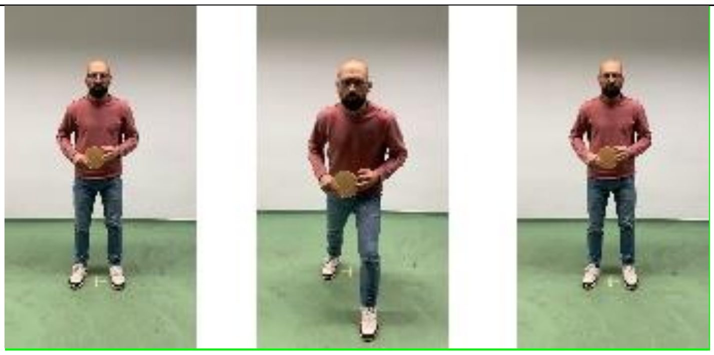
BACKWARD STEP LEFT	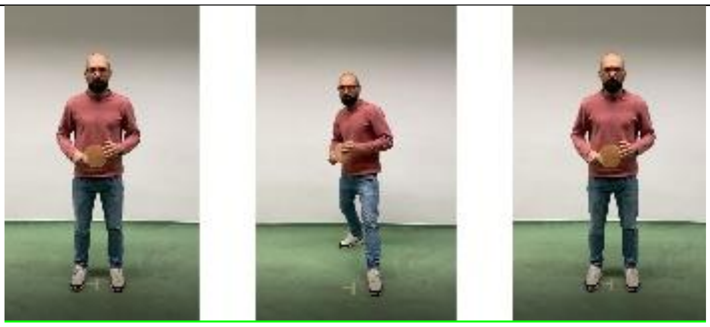
BACKWARD STEP RIGHT	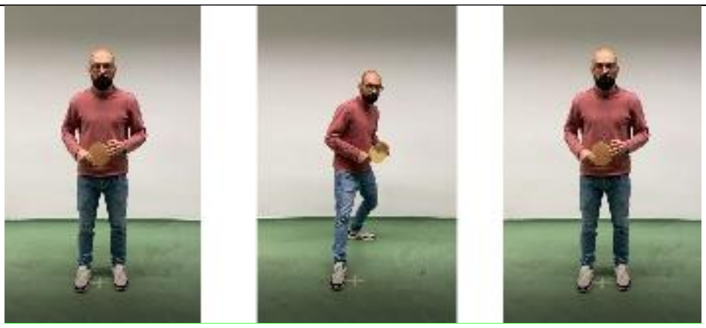
FOREHAND PLAY	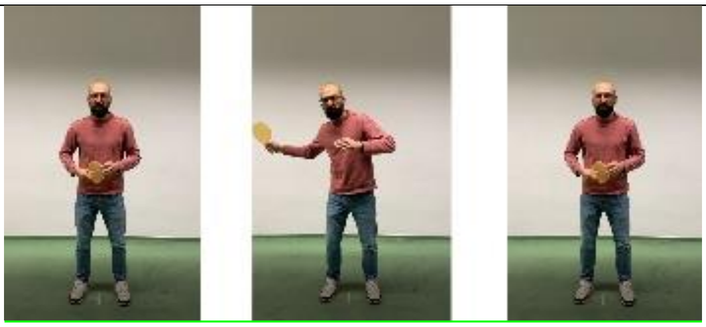

BACKHAND PLAY	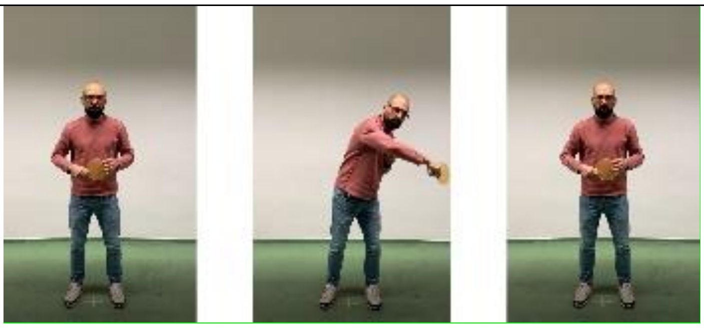
FORWARD STEP + FOREHAND	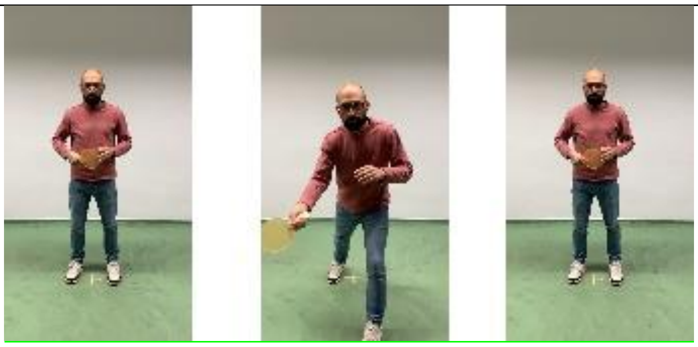
FORWARD STEP + BACKHAND	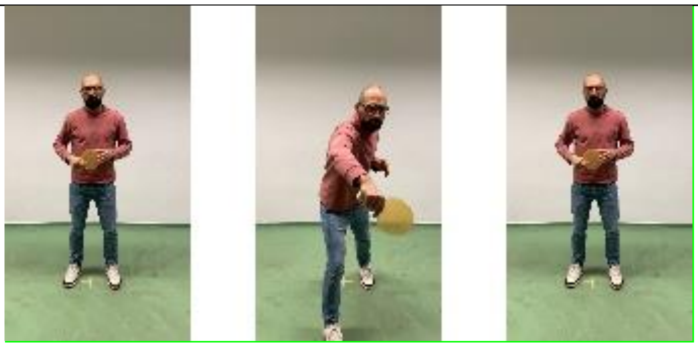

Head movements (20 repetitions for each of the 6 movement types, in pseudo-randomized sequence):

TILT LEFT	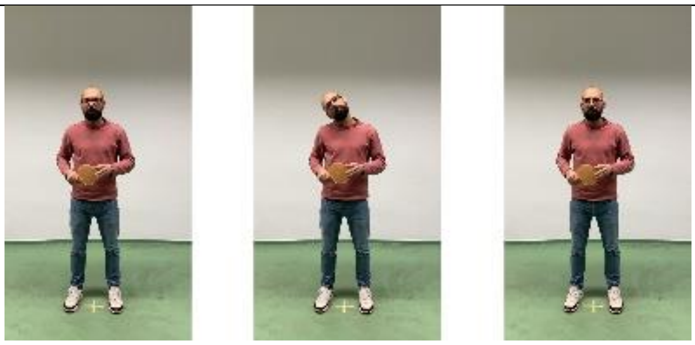

TILT RIGHT	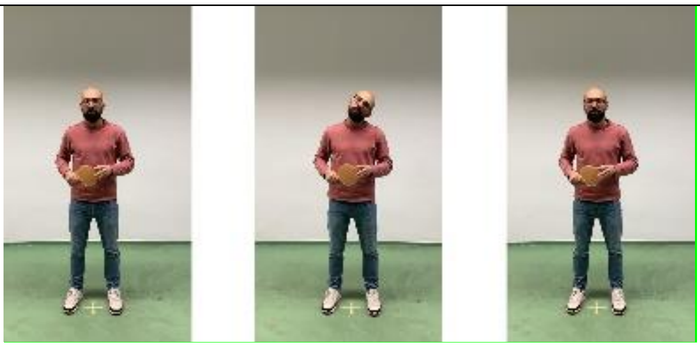
TILT FORWARD	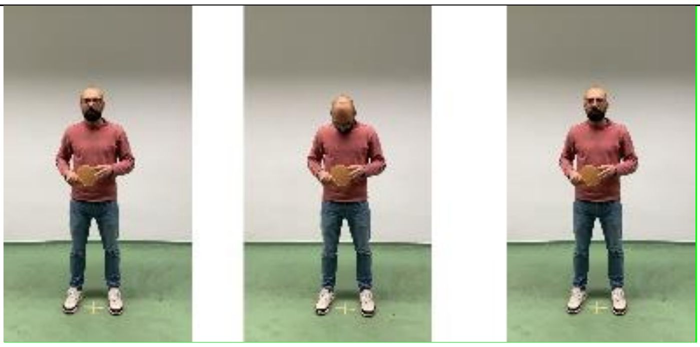
TILT BACKWARD	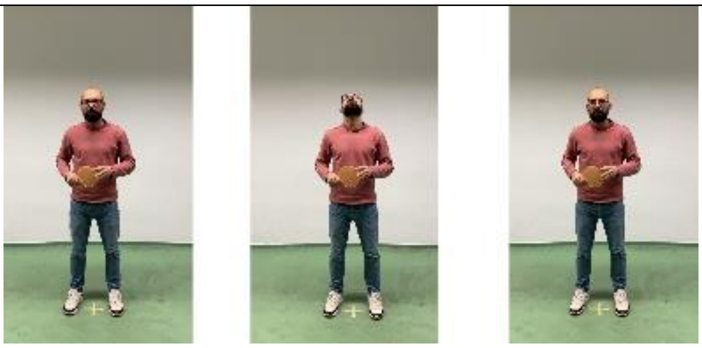
ROTATE LEFT	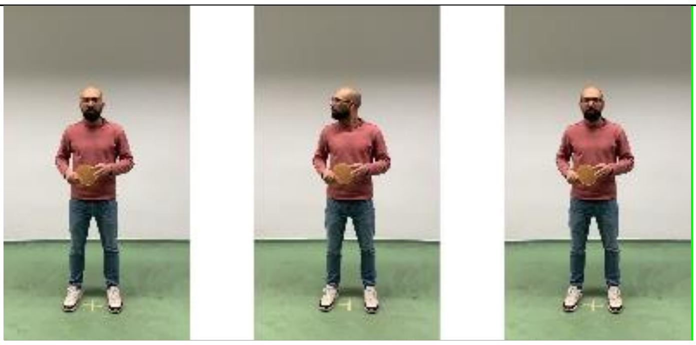
ROTATE RIGHT	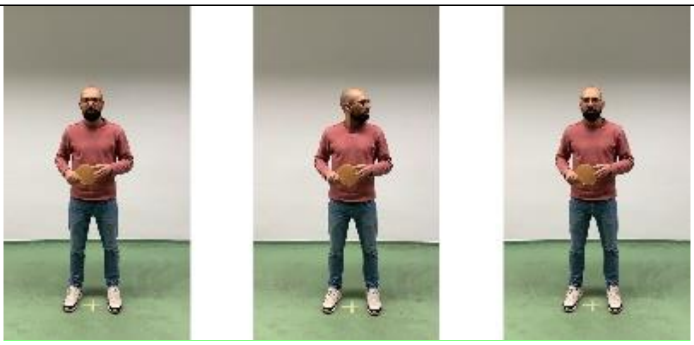

Movement instructions were presented by displaying the corresponding image for 3 seconds (as shown in the lists above), followed by an acoustic and visual cue to execute the displayed movement. Volunteers had 3 seconds to execute the movement prior to proceeding with the next instructions. Accordingly, 3 event codes were synchronously recorded with the EEG and accelerometer data, corresponding to a) image presentation, b) acoustic prompt, and c) visual cue to execute the movement.

The sequence of the two blocks of body and head movements was randomized. Moreover, three resting state acquisitions, each of 2 minutes duration, were interleaved with the movement blocks. An acquisition session consequently included 5 acquisition segments, structured as follows:

resting state + body movements + resting state + head movements + resting state

OR

resting state + head movements + resting state + body movements + resting state
